# Limited Mutation-Rate Variation Within the *Paramecium aurelia* Species Complex

**DOI:** 10.1534/g3.118.200420

**Published:** 2018-05-24

**Authors:** Hongan Long, Thomas G. Doak, Michael Lynch

**Affiliations:** * Institute of Evolution and Marine Biodiversity, KLMME, Ocean University of China, Qingdao, China 266003; † National Center for Genome Analysis Support, Indiana University, Bloomington, IN 47405; ‡ Biodesign Center for Mechanisms of Evolution, Arizona State University, Tempe, AZ 85287

**Keywords:** ciliated protozoa, mutation-accumulation, neutral evolution

## Abstract

Mutation is one of the most fundamental evolutionary forces. Studying variation in the mutation rate within and among closely-related species can help reveal mechanisms of genome divergence, but such variation is unstudied in the vast majority of organisms. Previous studies on ciliated protozoa have found extremely low mutation rates. In this study, using mutation-accumulation techniques combined with deep whole-genome sequencing, we explore the germline base-substitution mutation-rate variation of three cryptic species in the *Paramecium aurelia* species complex—*P. biaurelia*, *P. sexaurelia*, and *P. tetraurelia*. We find that there is extremely limited variation of the mutation rate and spectrum in the three species and confirm the extremely low mutation rate of ciliates.

Mutation is one of the main evolutionary forces driving genome evolution of organisms. Mutation accumulation (MA) techniques combined with deep whole-genome sequencing have greatly advanced genome-wide mutation-rate studies (Bateman 1959; Mukai 1964; Lynch *et al.* 2008). During MA, from a single-individual ancestor, multiple lineages are established and repeatedly single-individual transferred. Selection is greatly reduced by such strong population bottlenecks, and genetic drift plays the major role in the fate of mutations, allowing the random accumulation of most spontaneous mutations.

Although microbial eukaryotes represent the majority of eukaryotic species diversity (Green *et al.* 2004; Mcgrath and Katz 2004; Mora *et al.* 2011; Katz and Grant 2014) and have extremely divergent genome architecture (Lynch 2007), spontaneous mutations are notoriously difficult to study in these species. This is mostly due to the rarity of mutations (mutation rates in the range of 10^−12^ to 10^−10^ per nucleotide site per cell division), laborious culturing/transfers, lack of reference genomes, etc. As a consequence, genomic mutation rates of only a few microbial eukaryotes have been estimated directly (Lynch *et al.* 2008; Saxer *et al.* 2012; Sung *et al.* 2012; Zhu *et al.* 2014; Farlow *et al.* 2015; Ness *et al.* 2015; Long *et al.* 2016a; Long *et al.* 2016b; Hamilton *et al.* 2017; Long *et al.* 2018). An investigation of mutation-rate variation in microbial eukaryotes may help reveal mechanisms of mutation-rate evolution.

The *Paramecium aurelia* species complex is one of the best-studied model ciliates in genetics and epigenetics (Sonneborn 1975; Bétermier 2004; Aury *et al.* 2006; Beale and Preer 2008; Johri *et al.* 2017). This species complex consists of 14 cryptic species, which are morphologically indistinguishable from each other (Sonnerborn 1975). As with most ciliates, these species are binuclear, with a transcriptionally inert micronucleus (germline nucleus) and a transcriptionally active macronucleus (somatic nucleus) during vegetative growth. The micronucleus facilitates a unique MA opportunity in which mutations accumulate in a near-neutral manner (Sung *et al.* 2012; Long *et al.* 2013). This species complex also has the capacity for autogamous sexual reproduction, during which a whole-genome homozygote is developed from a single haploid germline genome. This greatly facilitates the localization of mutations accumulated in the germline micronucleus, which is otherwise transcriptionally silent during vegetative growth (and this also facilitates accumulating spontaneous mutations in an effectively neutral fashion). The germline mutations are amplified and made homozygous in the subsequent macronucleus after the autogamy process.

Species within the *P. aurelia* complex diverged >100 million years ago based on the known mutation rate in *P. tetraurelia* (Catania *et al.* 2009; Sung *et al.* 2012; Johri *et al.* 2017). These *Paramecium* species have different genomic G/C contents and genetic diversity (Aury *et al.* 2006; Catania *et al.* 2009; Johri *et al.* 2017), both of which might be related to differences in genome-wide mutational features, such as bias in the mutation spectrum—which is a strong evolutionary force in determining the genomic G/C composition across organisms (Long *et al.* 2018).

Here, we conducted a two-year mutation accumulation experiment on two closely-related species: *P. biaurelia* and *P. sexaurelia*. Together with the mutation rate previously reported for a related species, *P. tetraurelia* (Sung *et al.* 2012), we explore the base-pair substitution mutation-rate variation of the *Paramecium aurelia* species complex.

## Materials and Methods

### Strains, medium, and MA line transfers

We chose two species of the *Paramecium aurelia* complex for the mutation accumulation experiments: *P. biaurelia* V1-4 and *P. sexaurelia* AZ8-4. After two rounds of autogamy, single cells of the two strains were used to initiate the MA lines (48 lines for *P. biaurelia* and 32 for *P. sexaurelia*). MA lines were cultured in 150 µl Sonneborn’s *Paramecium* medium inoculated with *Klebsiella pneumoniae* subsp. *pneumoniae*. For each MA line—because a single cell could not always establish a new culture—we transferred eight single cells of each MA line to eight individual wells with medium on a 96-well plate using a dissection microscope; after two or three days, another eight single cells from the established culture were then transferred to wells and so on. Every three weeks, all MA lines were starved for five days to induce autogamy to renew the macronuclei. Autogamy was confirmed by Hoechst 33342 staining and fluorescence microscopy. In order to estimate the number of cell divisions between two consecutive transfers, total cells of each MA line after one culturing cycle were counted on a hemocytometer every month. The grand averages of cell divisions in one culturing cycle were 5.1 and 6.6 for *P. biaurelia* and *P. sexaurelia* respectively. In total, we conducted 156 and 148 transfers on average for *P. biaurelia* and *P. sexaurelia* respectively.

### DNA extraction, library construction, genome sequencing, and mutation analyses

After the final round of autogamy of all survived MA lines and manually washing off bacteria with 1× PBS on a dissection microscope (1× PBS paralyzes *Paramecium* cells which then stick to the bottom of Petri dishes, easing washing off food bacteria), we extracted DNA using the phenol-chloroform method. High-quality genomic DNA was then used to construct DNA libraries with the Nextera Sample Preparation Kit (Illumina). Size-selection for an insert size of 300 bp was performed and the libraries sequenced using HiSeq2500 2×150 rapid run at the Hubbard Center for Genome Studies, University of New Hampshire. After removing lines with low coverage or cross-contamination, 32 and 18 MA lines of *P. biaurelia* and *P. sexaurelia* respectively were used in the final mutation analyses. We followed Long *et al.* (2016a) for mutation analyses except that HaplotypeCaller in GATK 3.6 with standard hard filters (except MQ ≥ 60; all non-mutated lines support a different base) was used to call base-substitution mutations that only occurred in one MA line. All corresponding statistics were conducted in R 3.3.2. Read alignment for all mutation sites was validated visually with the Integrated Genome Viewer (IGV v. 2.3.5) (Thorvaldsdottir *et al.* 2013).

Equilibrium G/C composition by mutation pressure alone (*p_n_*) was calculated by following Lynch (2007):

$$p_{n}=\frac{u}{u+v},$$

where *u* is the mutation rate in the G/C direction (including A:T→G:C transitions and A:T→C:G transversions) and *v* is the mutation rate in the A/T direction (including G:C→A:T transitions and G:C→T:A transversions). Mutation bias in the G/C direction, *m*, was calculated by *m* = *u*/*v*.

### Data availability

Illumina sequence reads of MA lines in BAM format were deposited under a NCBI BioProject with accession number PRJNA394918, SRA No.: SRP113815. The authors state that all data necessary for confirming the conclusions presented in the article are represented fully within the article. Supplemental material available at Figshare: https://doi.org/10.25387/g3.6340790.

## Results and Discussion

From a single cell of each strain, we established 48 and 32 MA lines for *P. biaurelia* V1-4 and *P. sexaurelia* AZ8-4, respectively. Each MA line was transferred every two or three days and experienced periodic autogamy to avoid macronuclear senescence. The period of single-cell transfers (156 and 148 transfers on average, *i.e.*, ∼800 and 980 cell divisions; Supplemental Table S1) extended for 17 months. 34 and 21 lines survived to the end of this period.

**Figure 1:**
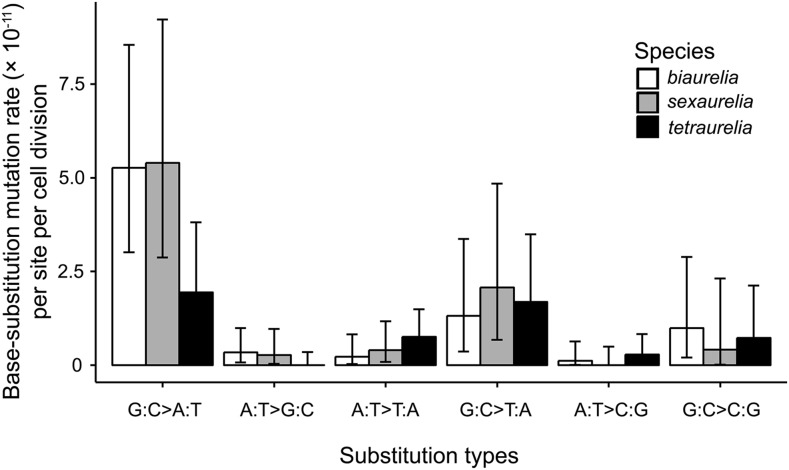
Mutation spectra of the three *Paramecium* species. Error bars are 95% Poisson confidence intervals.

For precise mutation analysis, we require MA lines with genome sequence coverage > 20× and without cross-line contamination. 32 and 18 final MA lines fulfilled these requirements for the final mutation analyses. Mean depth of coverage for the two species is 68× and 89× per line. After we filtered out reads that did not pass the quality filters, on average 62 and 85% of the two species’ MA line genomes were covered with high-quality/high-depth reads (Supplemental Material: Table S1). We detected 29 and 24 base-pair substitutions in *P. biaurelia* and *P. sexaurelia* respectively, yielding mutation rates of 2.44 × 10^−11^ (95% Poisson confidence interval 1.63–3.50 × 10^−11^, *P. biaurelia*; transition/transversion ratio 1.90) and 2.42 × 10^−11^ (1.55–3.61 × 10^−11^, *P. sexaurelia*; transition/transversion ratio 1.67) per nucleotide site per cell division, not significantly different from the previously reported *P. tetraurelia* mutation rate (Table 1). Most base substitutions are widely distributed across the whole genome on different scaffolds (Supplemental Table S2). 40% and 50% of all coding-region base substitutions are detected at the second position of codons for *P. sexaurelia* and *P. biaurelia* respectively (Supplemental Table S2). The base-pair substitutions are heavily biased in the A/T direction in all three species (Table 1; Figure 1). No significant difference for any specific type of mutations among the three species could be detected, neither does the mutation bias, which could result from the low statistical power of the small number of mutations in each species (Figure 1; Table 1). The limited number of mutations—even after 2–4 years of frequent single-cell bottlenecks—illustrate the difficulty of mutation-accumulation experiments using ciliates cultured in liquid medium. Automatic transferring operation is needed to finally resolve the mutation spectra of ciliates (currently, no ciliate species’ mutation spectrum has been resolved).

**Table 1:** G/C composition and mutation parameters of the three *Paramecium* species. CI: confidence intervals; G/C: genome-wide G/C composition, SE is binomial standard error; *m*, mutation bias in the G/C direction (calculaiton details are in the Materials and Methods section); *p4*: G/C content at fourfold degenerate sites; *p_n_*: equilibrium neutral expectation for G/C content by mutation pressure alone; S, population-scaled selection strength for G/C nucleotides at fourfold degenerate sites; µ, mutation rate × 10^−11^ per nucleotide site per cell division

Species	G/C (SE)	*m*	*p_n_*	*p4*	*S*	µ (95% CI)
*P. biaurelia*	0.25 (4.94×10^−5^)	0.05 (2.58×10^−2^)	0.06	0.20	1.37	2.44 (1.63, 3.50)
*P. sexaurelia*	0.24 (5.18×10^−5^)	0.04 (2.88×10^−2^)	0.03	0.14	1.89	2.42 (1.55, 3.61)
*P. tetraurelia*	0.28 (5.29×10^−5^)	0.08 (2.93×10^−2^)	0.07	0.24	1.26	1.94 (1.32, 2.84)

Despite the high species diversity of ciliates, very little research has directly addressed ciliate mutational processes (Sung *et al.* 2012; Long *et al.* 2013; Long *et al.* 2016b). Here we successfully accumulated dozens of base-pair substitutions in *P. biaurelia* and *P. sexaurelia*, supporting the conclusion that ciliates in general have extremely low germline base-substitution mutation rates (Sung *et al.* 2012; Long *et al.* 2016b). The consistently low germline mutation rates may be a consequence of the large effective population sizes of ciliates, which enhance the efficiency of selection for replication fidelity (Lynch *et al.* 2016), and/or the dimorphic nuclear structure, which expands the temporal period during which mutations are sequestered in a silent state within the germline (Sung *et al.* 2012).

The species in this study are known to have quite different π_s_ (the mean pair-wise nucleotide divergence between sequences in a population sample at fourfold degenerate sites, where any codon change will not change the coded amino acid; Table 1 in (Johri *et al.* 2017))(Table 3 in Catania *et al.* 2009): 0.0089, 0.0269, 0.0058 for *P. biaurelia*, *P. sexaurelia*, and *P. tetraurelia* respectively. Yet their mutation rates (*µ*) are quite similar, as discovered in this study. Since π_s_ = 4*N_e_*×*µ*, where *N_e_* is the effective population size (Nei and Li 1979), this implies that these species may have different effective population sizes.

As mentioned above, none of the three species’ mutation spectra were resolved. This hinders the attempts to explore the role of mutation bias in shaping the different genome G/C nucleotide compositions, as we have done in a wide variety of organisms with mutation spectra completely resolved (Long *et al.* 2018). Nonetheless, with the current mutation bias data of the three species, we calculated the equilibrium G/C contents and found that they are correlated with the observed G/C content, but not significant (*r* = 0.85, *P* > 0.05; Table 1). The lack of significance could be caused by only three data points or again the small number of mutations accumulated. Consistent with Long *et al.* (2018), we also found universal selective strength/gene conversion favoring high G/C content using the fourfold degenerate sites and equilibrium G/C content (equation 2 in Long *et al.* 2018) (Table 1). These findings thus confirm that the genome composition of ciliate species is also under heavy influence of mutations, though a complete mutation spectrum is needed to conclude on this.
